# Bone mineral density in partially recovered early onset anorexic patients - a follow-up investigation

**DOI:** 10.1186/1753-2000-4-20

**Published:** 2010-07-08

**Authors:** Ulrike ME Schulze, Simone Schuler, Dieter Schlamp, Peter Schneider, Claudia Mehler-Wex

**Affiliations:** 1 Department of Child and Adolescent Psychiatry/Psychotherapy, University of Ulm, Germany; 2 Department of Child and Adolescent Psychiatry and Psychotherapy, University of Würzburg, Germany; 3 Clinic for Nuclear Medicine, University of Würzburg, Germany; 4 Heckscher Clinic for Child and Adolescent Psychiatry, Munich, Germany

## Abstract

**Background and aims:**

There still is a lack of prospective studies on bone mineral development in patients with a history of early onset Anorexia nervosa (AN). Therefore we assessed associations between bone mass accrual and clinical outcomes in a former clinical sample. In addition to an expected influence of regular physical activity and hormone replacement therapy, we explored correlations with nutritionally dependent hormones.

**Methods:**

3-9 years (mean 5.2 ± 1.7) after hospital discharge, we re-investigated 52 female subjects with a history of early onset AN. By means of a standardized approach, we evaluated the general outcome of AN. Moreover, bone mineral content (BMC) and bone mineral density (BMD) as well as lean and fat mass were measured by dual-energy x-ray absorptiometry (DXA). In a substudy, we measured the serum concentrations of leptin and insulin-like growth factor-I (IGF-I).

**Results:**

The general outcome of anorexia nervosa was good in 50% of the subjects (BMI ≥ 17.5 kg/m^2^, resumption of menses). Clinical improvement was correlated with BMC and BMD accrual (χ^2 ^= 5.62/χ^2 ^= 6.65, p = 0.06 / p = 0.036). The duration of amenorrhea had a negative correlation with BMD (r = -.362; p < 0.01), but not with BMC. Regular physical activity tended to show a positive effect on bone recovery, but the effect of hormone replacement therapy was not significant. Using age-related standards, the post-discharge sample for the substudy presented IGF-I levels below the 5^th ^percentile. IGF-I serum concentrations corresponded to the general outcome of AN. By contrast, leptin serum concentrations showed great variability. They correlated with BMC and current body composition parameters.

**Conclusions:**

Our results from the main study indicate a certain adaptability of bone mineral accrual which is dependent on a speedy and ongoing recovery. While leptin levels in the substudy tended to respond immediately to current nutritional status, IGF-I serum concentrations corresponded to the individual's age and general outcome of AN.

## Background

Anorexia nervosa (AN) is an illness with major psychiatric and physical components - not simply a psychiatric condition - with a high risk of chronicity, complications, and adverse long-term effects. During childhood and adolescence, its specific psychopathology occurs at critical periods for bone growth and mineral accrual [1].

Mediated by nutritional deficits and hormonal abnormalities, peak bone mass - which should be completed around the age of 20 at least - may not be reached. Alterations in bone microarchitecture and persistent bone mineral deficiencies can follow, increasing the risk of osteoporotic fractures [2,3]. Lean body mass is a surrogate for muscle mass. While the nutrition-exercise-bone mass relationship in general is said to be complex, the enhancement of lean mass from long-term sports participation during adolescence results in greater bone mass accrual in healthy individuals [4]. Mechanical forces have been described as a factor in regulating bone modeling [5].

Deficient bone accrual is not limited to the acute phase of illness or the most severe stage of malnutrition. The linearity of association with disease duration and its mechanism are debated. Some authors suggest that adolescent AN shows a decreased bone turnover overall, in contrast to postmenopausal osteoporosis in which uncoupling of bone turnover is found, i.e. markers of increased bone resorption and decreased bone formation [6-10].

In premenopausal women, BMD determination alone is not adequate for assigning the labels "osteopenia" and "osteoporosis" (as per WHO guidelines). Thus, in this age group, labeling of changes should use terms like "poor bone mass accumulation" or "reduced bone mass". Furthermore, patients themselves may be better controls for follow-up examinations than age- and sex-matched healthy individuals [11].

Recovery of bone mineral density (BMD) in AN is described as a slow process [12]; it is a product of complex interactions between hormonal and nutritional factors [11]. Recent data suggest that bisphosphonates are effective in anorexic females [13]. However, at this stage, great caution is advised and - especially in premenopausal women - bisphosphonate use should be limited to clinical trials. This also applies to estrogen therapy in young women with AN because of the methodological problems and application technology involved. The results of different trials so far provide rather limited evidence [11,14,15]. Early detection of the illness and normalization of weight and menses are believed to be essential [16].

Leptin plays a key part in energy homeostasis [17]. Its serum levels correlate with body fat mass (FBM) and body mass index (BMI) both in healthy individuals [18], and in patients with AN [19]. In this patient group, weight loss-associated hypoleptinemia reflects both somatic and behavioral adaptations to starvation. The hormone also produces anorexigenic effects in the brain. It modulates the mesolimbic dopamine system via specific inhibitory neurons in the lateral hypothalamic area, decreasing feeding and body weight [20,21]. Secreted by fat cells and linking changes in body composition with bone formation and bone resorption, the cytokine-like hormone acts through its direct anabolic effects on osteoblasts and also through central effects (e.g. stimulation of the GH-IGF-1 axis, stimulation of beta2-adrenergic receptors, suppression of neuropeptide Y) [22,23]. However, we should note that both in-vitro or animal studies and human cross-sectional studies on the role of leptin in bone metabolism are not conclusive [24].

The IGF/IGF binding protein (IGFBP) system is known to be an essential component in the hormonal regulation of longitudinal growth. GH and IGF-I are involved in bone modeling and remodeling during childhood and adolescence; they are important regulators of bone homeostasis and one of the factors required to achieve normal longitudinal bone growth and bone mass. In adults, they are essential for bone maintenance [5]. The IGF-I present in the systemic circulation, synthesized by peripheral tissues, and expressed by osteoblasts regulates bone size, shape, and composition. Moreover, it plays a key role in adapting an individual's bone structure to mechanical loads during growth and development [5,25].

In patients with AN, abnormalities in the GH/IGF-I axis have a critical impact on the development of osteoporosis [5]. IGF-I as a nutritionally dependent bone trophic factor may be regarded as the major correlate of bone formation. Its serum concentration improves with weight gain: body mass index (BMI) is reported to show a positive correlation with free IGF-I. By contrast, independent of BMI, endogenous IGF-I, leptin, and androgen levels are suggested to predict bone microarchitecture [3].

Follow-up studies on BMD in anorexic patients with onset in childhood and adolescence are rare [26-29]; post-discharge histories in most cases are short or show great variation [30-33]. In particular, there still is a lack of prospective studies in this patient group. Despite the possibility of improving BMD in the long term [34], comparatively little is known about the effect of the general outcome of AN on bone accrual and hormonal correlations - especially with a view on former patients with an early onset of illness. Hormonal changes which lead to a normalization of essential bodily functions (e.g. resumption of menses) cannot be predicted by one specific (laboratory) parameter.

### Aims

The aims of our follow-up investigation were to assess the outcome of early onset AN, and its hormonally related effects on bone mineral parameters and their relation to lean body mass.

### Hypotheses

Because of the young age of our former patients and early manifestation of their restrictive AN, we hypothesized that bone mineral accrual would be comparatively deficient. We also postulated that a good global outcome and weight gain outcome would be able to counteract bone mineral loss.

### Expectations

We assumed a positive influence on bone mass development due to hormonal substitution, and we furthermore expected that moderate regular physical activity would have a positive effect on bone mineral density [27].

## Methods

We classified the extent of eating disorder symptomatology in accordance with generally accepted clinical outcome criteria [35-37]. As well, we expected baseline and follow-up data concerning BMD, bone mineral content (BMC), and the soft tissue composition of lean and fat body mass (LBM, FBM) to be related to clinical outcome.

In terms of an explorative substudy, we additionally estimated to see some association with serum leptin concentrations and IGF-I.

### Main Study

#### Participants

From an original sample of 103 inpatients, we reinvestigated BMD, BMC, LBM and FBM in 52 subjects (sample 1; for descriptive statistics see Table 1). Two of the former patients had died. A statistical comparison between participants and refusing individuals on age, duration of follow-up, BMI, duration of amenorrhea, BMD, BMC, LBM and FBM showed no significant differences except in the duration of amenorrhea before admission (5 months in participants vs. 8 months in refusing individuals). At follow-up, 23 individuals underwent an estrogen substitution, two of them less than 12 months.

**Table 1:** Sample description

*Main study/sample 1 (n = 52)*	*mean*	*SD*	*min*	*max*
Follow-up period (years)	5.23	1.69	3	9

*Age (t1/years)*	15.51	2.07	9.83	18.83

*Age (t2/years)*	20.78	2.72	12.80	26.70

*BMI (t1)*	14.74	1.88	10.42	18.67

*BMI (t2)*	20.13	2.79	14.26	28.14

				

***Sub study/sample 2 (n = 39)***	**mean**	**SD**	**min**	**max**

*Follow-up period (years)*	5.26	1.71	3	9

*Age (t1/years)*	15.47	2.10	9.83	18.83

*Age (t2/years)*	20.72	2.79	12.80	25.50

*BMI (t1)*	14.88	1.86	14.87	18.67

*BMI (t2)*	19.91	2.52	14.30	26.30

The patients, consecutively admitted, were reexamined by the first two authors after a post-discharge period of 5.2 years (± 1.7; range 3-9 years). All individuals met the DSM-IV diagnostic criteria for AN (restrictive subtype) upon admission [38]. Most of the patients had been hospitalized initially for AN. Male patients were excluded because of the small number of patients and the effects of hormones on bone development. There were no additional inclusion or exclusion criteria.

#### Procedures

The study was reviewed by the appropriate institutional review board. All patients gave informed consent prior to their inclusion into the study. We did not measure the baseline values of the nutritionally dependent hormones.

The initial DXA measurement (t1) was performed within four weeks of admission. At follow-up (t2), current body composition was compared with baseline, and to provide comparability with previous studies of our study group [39,40], the same reference data were used [41-44].

At follow-up, the outcome criteria included the current body mass index, presence or absence of menstrual cycle, and/or bulimia nervosa (see Table 2). The general outcome resulting from these factors (predominantly physical parameters, but also bulimic symptoms in cases of poor outcome) was defined according to the criteria of Morgan and Russell, modified by Ratnasuriya [35-37].

**Table 2:** General outcome - sample 1 (n = 52)

*Outcome*	*n* *(%)*	*BMI U1* ***(kg/m^2^*)**	*BMI U2* ***(kg/m^2^*)**	*amenorrhea* *(months)*
*Good* (BMI ≥17.5; regular menstrual cycle)	**26 **(50.0%)	14.9 (± 1.9)	20.5 (± 2.2)	13 (± 15)

*Intermediate* (BMI < 17.5 or BMI > 26; *or *irregular menstrual cycle/fluctuations of weight, amenorrhea)	**20 **(38.5%)	15.1 (± 1.9)	20.6 (± 3.0)	32 (± 27)

*Poor* (BMI < 17.5; amenorrhea; bulimic symptoms)	**6 **(11.5%)	13.1 (± 1.9)	17.1 (± 2.6)	27 (± 16)

Hormone replacement therapy was assumed if estrogens had been taken without interruption for at least 12 months before follow-up.

Participants who had exercised consistently for at least 9 months during the follow-up period were classified as physically active.

#### Instruments

The specific eating disorder psychopathology at follow-up was assessed using a clinical semi-structured interview and the CIDI [45]. These instruments allowed the classification of specific symptoms and their severity in cases of persistence or manifestation of an eating disorder (anorexic or bulimic symptoms).

#### Physical examination

All participating patients underwent a physical examination to ensure an overall physical assessment.

#### Laboratory tests

In addition to the physical examination, we carried out a laboratory assessment using commercially available tests for the blood count, electrolyte balance, and pancreatic, liver, kidney, thyroid and gonadal function.

#### Bone and lean body mass parameters

Dual-energy x-ray absorptiometry (DXA, LUNAR DPX-L, Lunar Corporation, Madison, USA) was used to perform whole body scans. These scans recorded the total body mineral content BMC [kg], BMC projected on bone area (which is commonly defined as BMD [g/cm^2^]), lean body mass LBM [kg], and fat mass FBM [kg]. Generally, BMD depends on the bone area projected on a plane. Bone area adds a blurring parameter to bone mineral content as the primary DXA finding.

#### Analysis

Statistical analysis was made using SPSS™. Student's t-test for dependent samples was used for the bone mineral parameters. The Kruskal-Wallis test was applied to differentiate between the three outcome groups (good, intermediate, and poor outcome), while allowing for the small sample size. We used the Mann-Whitney test for the differences between any two groups. The significance level was set at: *: p < 0.05; **: p < 0.001. The Least Significance Difference group test (LSD) for unequal (and small) sample sizes was used to test group range differences in the BMC z-score changes, as well as changes in the ratio of BMC/LBM. The significance level was set at *p *< 0.05. The z-scores were calculated using a polynomial fit function based on the means and standard deviations of the normal BMC values from Zanchetta et al. [44].

### Substudy

#### Participants

A subgroup of 39 participants (sample 2) also agreed to give an additional blood sample at follow-up (for descriptive statistics of both samples see Table 1).

#### Nutritionally dependent hormones

We determined the serum levels of IGF-I, and leptin by in-house radioimmunoassays, described previously [18,46-49]. IGF-I was measured by IGFBP-blocked assay in the presence of a large excess of IGF-I to inhibit the interference of binding proteins. The serum was always obtained in the morning.

#### Analysis

Spearman's correlation coefficients were calculated between serum leptin levels and IGF-I, and BMD, BMC or body composition (FBM, LBM) changes.

## Results

Follow-up examinations were performed 5.2 years (± 1.7; range 3-9 years) after discharge. The median age of our patients at time of first examination was 15.5 years (± 2.1; range 10-19) and 20.8 years (± 2.8; range 13-26) at follow-up.

Mean BMI had increased from 14.7 kg/m^2 ^(± 1.9) to 20.1 kg/m^2 ^(± 2.8). Lean body mass of our patients increased from 34 (± 5) to 39 (± 4) kg during the post-discharge period.

### Main study

#### General outcome of AN

50% (n = 26) of the post-discharge sample presented a good general outcome (Table 2). At follow-up, 6 individuals (11.5%) suffered from anorexia or bulimia nervosa. Duration of amenorrhea considerably differed (13-32 months; Table 2). Detailed information concerning our former patients with a poor outcome is given on Table 3.

**Table 3:** Detailed information concerning the patients with a poor outcome - sample 1 (n = 52)

*Individual Person*	*Follow-up period* *years*		*Age (t2)* *(years)*		*BMI (t1)* ***(kg/m^2^*)**		*BMI (t2)* ***(kg/m^2^*)**		*amenorrhea* *(months)*	*Binging/purging*
*A*	4		19.5		14.5		16.4		14	yes

*B*	3		20.6		10.8		22.1		24	yes

*C*	4		19.5		12.8		16.6		36*	no

*D*	4		20.3		12.5		14.3		32*	no

*E*	3		19.5		13.9		17.0		4*	no

*F*	7		21.7		14.4		16.5		50	yes

* Hormone resp. estrogen substitution at follow-up

#### Physical examination

In the general physical examination, 35% of the former patients showed dermatologic signs of AN such as acrocyanosis or lanugo hairs. Tanner stages were appropriate for the individual age.

#### Laboratory tests

A low-T3 syndrome was found in 8 patients; 2 participants suffered from hypothyroidism; in 19 cases, a slight increase of amylase was observed. There were no further pathological findings.

#### Bone and lean body mass parameters

For the entire post-discharge sample (n = 52), all essential body composition and bone mineralization parameters (FBM, LBM, BMC, BMD) exhibited a considerable accrual (Table 4). An association between the differences and general outcome of AN was shown (Table 5). BMC z-score changes are illustrated in Table 6.

**Table 4:** Body composition and bone parameters at first examination (t1) and follow-up (t2) - sample 1 (n = 52)

Parameter	t 1	t 2
**Fat Body Mass (FBM) (%)**	12 (± 7)	25 (± 8)*

**Lean Body Mass (kg)**	34 (± 5)	39 (± 4)*

**Bone Mineral Content (kg)**	2.04 (± 0.34)	2.37 (± 0.32)*

**Bone Mineral Density (g/cm^2^)**	1.04 (± 0.08)	1.09 (± 0.07)*

*p < 0.001 (t-test for dependent samples)

**Table 5:** Bone mineral mass and body composition (means/SD) according to the general outcome - sample 1 (n = 52), p-values refer to a t-test for dependent samples and indicate differences between first examination and follow-up

*Outcome*	*good* *n = 26*	*intermediate* *n = 20*	*poor* *n = 6*
**BMI (kg/m^2^)**	20.5 (± 2.2)	20.6 (± 3.0)	17.1 (± 2.6)
**FBM (%)**	27 (± 8)** p < 0.001 (+)	25 (± 8)** p < 0.001 (+)	19 (± 7)* p = 0.021 (+)
**Δ FBM (Mean/SD)**	0.13 (± 0.10)	0.11(± 0.07)	0.12 (± 0.10)
**LBM (kg)**	39 (± 5)** p < 0.001 (+)	39 (± 3)* p = 0.001 (+)	36 (± 4) *p *= 0.062 (+)
**Δ LBM (Mean/SD)**	4.94 (± 6.5)	3.90 (± 3.48)	4.27 (± 4.28)
**BMD (g/cm^2^)**	1.10 (± 0.07)** p = 0.000 (+)	1.09 (± 0.06) *p *= 0.050 (+)	1.02 (± 0.08) *p *= 0.125 (-)
**Δ BMD (Mean/SD)**	0.08 (± 0.07)	0.02 (± 0.05)	-0.03 (± 0.04)
**BMC (kg)**	2.43 (± 0.30)** p < 0.001 (+)	2.38 (± 0.3)** p <0.001 (+)	2.04 (± 0.3) *p *= 0.872 (+)
**Δ BMC (Mean/SD)**	0.08 (± 0.07)	0.02 (± 0.05)	-0.05 (± 0.04)
**BMC/LBM (at follow-up)**	0.062 (± 0.008) *p *= 0.15	0.060 (± 0.007) *p *= 0.53	0.054 (± 0.004)* p < 0.05
**Δ BMC/LBM (Mean/SD)**	-0.003 (± 0.008)	-0.001 (± 0.008)	+0.007 (± 0.004)

+ = increase, - = decrease

* p < 0.05; ** p < 0.001

**Table 6:** BMC z-score-changes and differences according to the general outcome and in relation to each other (Least Significance Difference Group Test) - sample 1 (n = 52)

Outcome BMC z-core according to the outcome	Good M = .399	Intermediate M = .166	Poor M = .166
**Good**		0.32	0.02

**Intermediate**	0.32		0,11

**Poor**	0.02	0.11	

**Figure 1:**
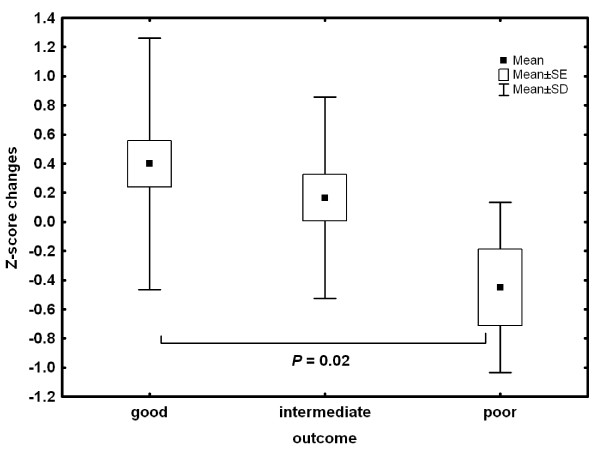
**BMC z-score changes differentiating between three groups**.

**Figure 2:**
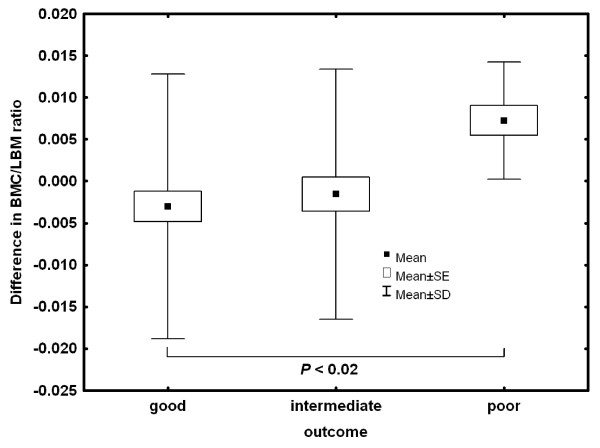
**Differences in the ratio of bone mineral mass and lean body mass at follow-up**.

The z-score changes in BMC values were significantly different among the three outcome groups (good vs. poor: *p *= 0.02; figure 1), whereas LBM accrual showed no significant differences. However, the ratio of BMC to LBM changes was significantly different between the three outcome groups (good vs. intermediate: *p *< 0.05, good vs. poor: *p *< 0.02; figure 2). At follow up, the BMC to LBM ratio had not changed in the good and intermediate group over time, but there was a significant deficiency in BMC accrual in the poor outcome group (*p *< 0.05, Table 5).

#### Physical activity

A single sport or various kinds of sports (9-month minimum) were reported by 26 (50%) of the re-investigated individuals. Of these, 23 had a BMI ≥17.5 kg/m^2 ^at follow up. There was no significant association between regular physical activity and weight restoration (BMI ≥17.5 kg/m^2^) (χ^*2 *^= 0.165; df = 1; p = 0.69). While 16 out of 26 (61.5%) participants with a good outcome reported that they worked out regularly, 13 of 20 (65%) individuals with an intermediate outcome did not (χ^*2 *^= 3.185; df = 2; p = 0.20). Merging both groups to compare them as one with the poor outcome group, did not reveal any statistically relevant differences.

#### Hormone replacement

32 patients reported taking hormone Tablets - predominantly in the form of contraceptives - for at least 12 months. Effects of hormone replacement therapy are given in Table 7.

**Table 7:** Effects of hormone replacement at follow-up (Mean/SD), p-values refer to a t-test for dependent samples and indicate differences between first examination (t1) and follow-up (t2) - sample 1 (n = 52)

	*BMD* *(g/cm^3^)*	*BMC* *(kg)*	*LBM* *(kg)*	*FBM* *(%)*
**Hormone replacement**	1.10 (± 0.06) p < 0.001 (+)	2.40 (± 0.3) p < 0.001(+)	39 (± 5) p < 0.001(+)	26 (± 9) p < 0.001(+)
**Without substitution**	1.06 (± 0.07) p = 0.028 (+)	2.31 (± 0.4) p = 0.001 (+)	39 (± 4) p < 0.001(+)	25 (± 6) p < 0.001(+)
**p-level**	0.06	0.33	0.96	0.65

#### Correlations

The duration of amenorrhea correlated negatively with BMD (r = -.362; *p *< 0.01). While regular physical activity tended to result in a gain only of BMD (- 0.063 (± 0.07) compared with 0.028 (± 0.07)), BMC showed a positive correlation with regular work-outs. Hormone replacement did not have a distinct influence on bone development (Table 7). Unlike the significant group differences in BMD baseline values (t1), the differences at follow up did not reach statistical significance.

### Substudy

#### General outcome of AN

The 39 participants who agreed to an additional blood taking were classified as follows: 20 (51.3%) showed a "good" outcome, whereas 15 (38.5%) individuals had an "intermediate", and 4 (10.3%) a "poor" general outcome according to the Morgan and Russell method, modified by Ratnasuriya [35-37].

#### Nutritionally dependent hormones

Serum leptin levels varied from 1.48 μg/l (the BMI of this patient was 17.2 kg/m^2 ^over several years) to 43.1 μg/l (correlated with a current BMI of 22.1 kg/m^2 ^- this patient had been anorectic for years and her current symptoms had changed to binge eating with a corresponding weight gain of 15 kg over 2 months).

Using age-related standards (Blum, 1996) to assign the mean values of insulin-like growth factors and its binding proteins, IGF-I levels below the 5^th ^percentile were found.

Table 8 shows the analyzed bone and body composition parameters according to outcome group. The Kruskal-Wallis test revealed significant differences between the groups in BMD (p = 0.03), BMC (p = 0.03) and IGF-I (p = 0.02). In terms of trend, a better outcome group status resulted in higher BMD, BMC and IGF-I values.

**Table 8:** Bone mineral density, body composition and nutritional dependent hormones according to the general outcome - sample 2 (n = 39; Means/SD)

	*Good* *(n = 20)*	*intermediate* *(n = 15)*	*poor* *(n = 4)*	*X^2^* *(df = 2)*	*p*
**BMI (kg/m^2^)**	20,16 (± 2.10)	20,26 (± 2.10)	17.34 (± 3.37)	3.97	0.14
**BMD (g/cm^2^)**	1,10 (± ,071)	1,08 (± ,059)	0.99 (± 0.02)	7.27	0.03*
**BMC (kg)**	2.37 (± 0.31)	2,35 (± 0,32)	1.92 (± 0.14)	7.28	0.03*
**FBM (%)**	26.85 (± 7.64)	22.76 (± 7.46)	19.53 (± 8.61)	3.64	0.16
**LBM (kg)**	38,28 (± 4,97)	39,16 (± 3,93)	35,37 (± 3,93)	3,47	0.18
**Leptin (μg/l)**	12,36 (± 12,44)	5,37 (± 2,63)	12.40 (± 20.48)	3.54	0.17
**IGF-I (μg/l)**	216,45 (± 80,63)	223,07 (± 71,09)	119.75 (± 37.77)	7.72	0.02*

* = p < 0.05

#### Correlations

Correlations were found between serum leptin concentrations and current body composition parameters. Correlations between physical status and serum and bone parameters at follow-up are given in Table 9. Specifically, there were significant correlations between leptin and BMI, leptin and BMC, and leptin and FBM. Moreover, correlations were found between LBM and BMI, and LBM and BMC.

**Table 9:** Correlations (Spearman): body composition, bone and serum parameters - sample 2 (n = 39)

	Leptin	IGF-I	FBM	LBM	BMI	BMC
**age (t2)**	-0.30	-0.42**				

**BMI **(body mass index)	0.62**	-0.08		0.47**		0.52**

**FBM **(fat body mass)	0.85**	-0.12			0.68**	0.49**

**LBM **(lean body mass)	0.21	0.15			0.47**	0.60**

**BMC **(bone mineral content)	0.35*	0.11		0.60**	0.52**	

**BMD **(bone mineral density)	0.15	0.28			0.36*	0.73*

**IGF-I**	**-**0.04	1.00				

**Glucose**	0.48**	-0.15	0.54**	0.35*	0.42**	

* = *P *< 0.05

** = *P *< 0.01

## Discussion

Along with assessment of the general outcome of 52 former anorexic inpatients, we tracked their bone mineral development in a follow-up investigation. We also investigated possible interactions between bone mineral parameters and nutritionally dependent hormones (leptin, IGF-I) in a subgroup of 39 individuals to test the possible impact of nutritional status on bone development.

In agreement with other follow-up studies [50-52], we found a good general outcome after 5.3 years in half of the post-discharge group (Table 2).

At first glance and conditionally contrary to our initial expectations, bone mineral accrual was positive over the entire post-discharge sample (Table 4). More explicitly considered - and this was replicated in other studies - clinical improvement correlated positively with both BMC and BMD [28,53].

A good outcome of AN was associated with a small increase in BMC z-scores (Figure 1). This is of some importance, because results of a previous study on the body composition of 31 anorexic adolescent girls demonstrated that BMC reflects the bone/muscle relationship better than BMD [39]. A poor outcome seems to be associated with a tendency toward bone loss. In spite of a possible catch-up effect associated with a good short-term outcome [31] and a long-term absence of clinically relevant symptoms of AN, complete normalization of BMD cannot be necessarily expected [33].

Weight restoration and resumption of menses both are known to be the most important preconditions for bone recovery [16]. In particular, the results of a prospective observational study on 34 girls with AN (aged 12-18 years) revealed that the recovery of menses is an essential pre-condition for stabilization of BMD measures [28].

In anorexic individuals, weight gain represents a significant and independent predictor of BMD, whereas adipose tissue is suggested to play a substantial role in the recovery of menstruation cycles (due to co-enabling ot a hormonal "reset") and weight-related protective effects on bone [54]. In our sample, fat mass revealed a significant increase, and the duration of amenorrhea correlated negatively with BMD, but not with BMC. Though, weight gain alone appears not to be sufficient to explain any increases in BMD. In a re-investigation of a small sample of anorexic adult patients, Baker et al. [55] showed that behavioral factors such as vomiting, nicotine and alcohol intake also may predict a reduction of BMD.

Amenorrhea in this patient group is thought to be an adaptive response to an energy deficit, partially mediated by leptin. As treatment with estrogen replacement therapy does not reverse the bone loss, the administration of recombinant leptin in order to restore LH pulsatility and ovulatory menstrual cycle may be worth considering prospectively [56] - but cautiously because of its anorexigenic effects [20,21]. On the other hand, in our sample as in several other studies, hormone replacement therapy did not show a distinct effect on bone mineral development [57,58].

Excessive exercise in patients with AN may be correlated with increased psychopathology. It plays an important role in the progression of the disease by accelerating weight loss during dietary restriction. However, and also in our sample, a *moderate *extent of physical activity tends to result in a protection of further bone mineral development [59,60].

An evaluation of long-term (negative) effects on BMD in 87 women diagnosed with menstrual disorders during adolescence revealed a restrictive eating disorder at follow-up (six years after initial assessment) as the strongest predictor of low BMD, whereas a BMI > 22 and high physical activity appeared to be the most important counter-indicators [32]. Particularly with regard to bed rest, a pilot study on patients aged 13-21 years showed that limitation of physical activity during hospitalization for patients with AN is associated with suppressed bone formation and resorption and an imbalance in bone turnover [61]. These results agree with those on BMD in male adolescents with AN. A follow-up study on 20 patients revealed that < 3 hours/week of physical activity correlated strongly with osteopenia [62].

In this context, lean mass may have a functional impact. There are rare findings that the ratio between lean body mass and bone mineral mass accrual (BMC/LBM) is also compromised [63]; BMC/LBM curves always correlate linearly. Multiple regression tests have shown little or no independent interaction of body weight or height with those relationships [64].

In a study on 24 adolescent patients with AN, Wong et al. found significant correlations between lean mass and BMC/BMD, but no reduced bone mass [65], whereas our results clearly indicate that BMC decisively developed a deficiency in relation to LBM. (This observation is independent of z-scores or t-scores based on any suitable reference population.)

The clinical heterogeneity of our sample is reflected in the wide variation in serum leptin levels and presumably also their comparatively high mean values, especially in the poor outcome group - maybe partially caused by frequent changes in eating behavior (Tables 3 and 8). Current nutritional status has a possible equivalent in individual serum glucose levels. Here, a correlation with leptin was also found (Table 9), which may be a possible dynamic component of glucose regulation [66,67]. Both leptin and glucose correlated significantly with BMI and FBM, but LBM also correlated with serum glucose (Table 9).

Long-term and sustained weight recovery may be necessary before significant improvements are observed [7]. Findings in the literature for body mass index [68] suggest that a BMI threshold value (16.4 kg/m^2^) may correlate with positive effects on bone formation.

Appraisals on leptin and bone development especially in premenopausal women are rare and mostly limited to bone turnover markers [69]. In their study on 19 anorexic inpatients, Heer et al. [70] associated a nutritionally induced increase of IGF-I and leptin concentrations ("nutritional rehabilitation") with a possible and direct effect on bone formation. While there was a correlation between leptin and BMC also in our sample, we could not demonstrate a strong association between IGF-I and bone mineral parameters (Table 9) [71,72]. Nevertheless, in accordance with a reported age-related decline of IGF-I, a negative correlation between IGF-I and age was found (Table 9) [73].

In terms of the entire group and using age-related standards, our patients showed low serum IGF-I concentrations. However, there is an obvious difference between outcome groups with poor outcome associated with the lowest IGF-I levels and vice versa. These findings agree with those of Legroux-Gerot et al., who investigated a group of 113 women 5.7 years after eating disorder onset [74]. Due to undernutrition, acquired GH resistance and decreased somatostatinergic inhibition in this patient group must be assumed [75,76].

Taken together, our results suggest a relatively direct adaptation of leptin activity to weight gain, body composition (BMI, FBM), and subsequently to BMC changes. Serum leptin levels significantly correlated with nutritional status parameters (BMI, FBM, glucose levels), whereas IGF-I serum concentrations did not.

Recovery status of anorexic patients does not only refer to the actual body weight or resumption of menses, especially in individuals who are on contraceptives. In the clinical context, we should carefully look on psychopathological details (concerning eating disorder specific core beliefs and possible comorbid conditions, e.g. depressive or anxiety disorders), assessed by personal exploration and also by standardized instuments. Based on a trusting relationship between patient and therapist, especially symptoms of social anxiety and the current individual impact of the eating disorder itself also should be asked for.

Limitations of our study include no assessment of bone turnover markers, the heterogenity of the group at the time that mainly the hormones were measured, and our comparatively small sample size. Subgroups should have had additional explanatory power to provide a more differentiated look at eating disorder outcome-associated changes in bone metabolism. We have also not collected data on nicotine or alcohol consumption.

## Conclusions

Our hypotheses concerning the association between the general outcome of AN during childhood and adolescence and bone mass accrual could be confirmed. With a view to future trends, and also in accordance with Wentz et al., a long-term BMD catch-up is something to hope for [34].

In our sample, bone mass accrual was clearly related to weight gain and resumption of menses. This agrees with the results of other studies on teenage onset anorexic patients [27-29,34]. While hormone replacement did not induce distinct positive effects on bone mineral development, regular physical activity did it by trend [27]. There was no direct association between athleticism and the amount of weight restoration (BMI ≥ 17.5 kg/m^2^).

Reduced BMD should not be seen as the decisive indicator of deficient bone development due to AN, especially in the poor outcome group. The different grades of recovery from AN, also dependent on the nutritional state of the participants, primarily seem to have an essential impact on the ratio of BMC to LBM. Thus, a deficient imbalance in bone mass in relation to muscle mass (which is a surrogate for muscle strength) during AN, may be the cause of an increased bone fragility in later life [77].

The interactions between the nutritional status of former anorexic patients, the re-accrual of their bone mass, and the serum concentration of nutritionally dependent hormones seem to be very complex. Although the results of a longitudinal study on 42 adolescent anorexic females by Jagielska et al. [78] reveal an improvement in BMC and BMD which was clearly related to the nutritional status, the mechanisms underlying bone loss in AN patients still remain unclear. Despite of the predictive value of hormonal and nutritional hormones (estrogen, insulin-like growth factor, leptin), our detailed knowledge about their correlations with bone mineral development in anorexic patients still remains unsatisfactory [79,80]. Based on this rationale, and in order to develop a more profound understanding of these processes and their possible influence on developing bone microarchitecture, we should carry on measuring bone mineral and body composition parameters parallel to (nutritionally dependent) hormones. Further realization is particularly supposable within the framework of interdisciplinary elaborated clinical studies, with an intended impact on future therapy.

Leptin actions seem to adjust rapidly to current changes, whereas results for IGF-I may indicate some longer-lasting aberrance in hormonal functions due to pathologic eating behavior. Nevertheless and propably partly linked to the individuals amount of body fat mass, both nutritionally dependent hormones are suggested to be essential components of a preservation of neuroendocrine control of reproductive function, also in a subset of patients who maintain menses despite low weight [81].

The individual healing process in anorexia nervosa requires a certain sustainability of (behavioral and psychopathological) change, and not just physical stablization. Therefore, with a view to a sustainable impact and validity of clinical outcomes on bone mass development, clinicians should preferably strive for an all-out and long-lasting stabilization in their anorexic patients and should not merely focus on weight normalization. The key to effective treatment of early onset anorexia nervosa is the early detection of disease and an evidence-based treatment approach aiming for recovery as an enduring and global process involving both psychological and physiological normalization.

## Competing interests

Except for essay analysis (see below), none of the researchers was paid any salary or received any financial support from any commercial source for this research.

## Authors' contributions

All authors have contributed essential parts to the manuscript and are entirely responsible for its scientific content. PS and DS planned the study. US, SS and PS carried out the investigation. US and PS created and edited the drafts, SS and PS did the main part of data analysis. CMW revised the draft critically. All authors approved the final manuscript.
